# The Medical Student’s Vade Mecum

**Published:** 1857-12

**Authors:** 


### BOOK NOTICES.

THE MEDICAL STUDENT'S VADE MECUM: A Compendium of Anatomy, Physiology, Chemistry, Poisons, Materia Medica, Pharmacy, Surgery, Obstetrics, Practice of Medicine, Diseases of the Skin, etc. etc. By GEORGE MENDENHALL, M. D., Professor of Obstetrics and Diseases of Women and Children in the Ohio Medical College, Member of the American Medical Association, etc., etc. Lindsay & Blakiston, Philadelphia, 1857, pp. 692. For sale by Keen & Lee, Chicago.

It will be seen by the title and date of Dr. Mendenhall's book, that it is extremely comprehensive and posted up to the times, containing the whole science of medicine in 692 pages, and issued in 1857.

Considering that the doctor is occupied for a good portion of the year in oral teaching, we cannot believe that he would make the gigantic effort necessary to compress so large a mass into so small a space, were it not a paying process. As it is, we think it but right and proper to congratulate Dr. M. on his success in both these respects. We suppose books of this sort act on the principle of compressed sponge, when once fairly crammed into the head of the student, they expand to such an extent, as to amplify his learning to the degree of mental saturation. Thus, other more voluminous and valuable works are dispensed with, at least, in most cases. Seriously, we think, they have a bad influence, and would prefer that they would never be published. We believe, however, that Dr. Mendenhall's book is equal, if not superior, to anything of the kind we have ever seen.

